# The N-shaped environmental Kuznets curve: an empirical evaluation using a panel quantile regression approach

**DOI:** 10.1007/s11356-017-0907-0

**Published:** 2017-12-12

**Authors:** Alexandra Allard, Johanna Takman, Gazi Salah Uddin, Ali Ahmed

**Affiliations:** 1 Department of Management and Engineering, Linköping University, 581 83 Linköping, Sweden; 2 Swedish National Road and Transport Research Institute, Teknikringen 10, 114 28 Stockholm, Sweden

**Keywords:** CO_2_ emissions, Renewable energy, Trade, Institutions, Quantile regressions

## Abstract

We evaluate the N-shaped environmental Kuznets curve (EKC) using panel quantile regression analysis. We investigate the relationship between CO_2_ emissions and GDP per capita for 74 countries over the period of 1994–2012. We include additional explanatory variables, such as renewable energy consumption, technological development, trade, and institutional quality. We find evidence for the N-shaped EKC in all income groups, except for the upper-middle-income countries. Heterogeneous characteristics are, however, observed over the N-shaped EKC. Finally, we find a negative relationship between renewable energy consumption and CO_2_ emissions, which highlights the importance of promoting greener energy in order to combat global warming.

## Introduction

Global warming has become one of the most serious world problems today (Duan et al. 2016). During the Paris Climate Conference in 2015, officially known as the 21st Conference of the Parties (COP21), several goals for keeping the rise in global temperature well below 2° were set up (United Nations 2017). In order to combat climate change issues alongside economic prosperity and to reach the COP21 goals, it is important to understand the effect of economic growth on the environment. Environmental degradation can have devastating consequences for humanity, such as health impacts, floods, droughts, damage to ecosystems, and adversely affected economic growth (IPCC 2014). At the same time, human activity is the main driving force behind climate change (Steffen et al. 2011).

In the environmental economics literature, the relationship between environmental degradation and economic growth is well known as the environmental Kuznets curve (EKC). The EKC suggests that environmental degradation initially rises with per capita income. However, with economic growth comes an increased demand for environmental quality, leading to a decreasing environmental deterioration (Hussen 2005). If there is an inverted U-shaped EKC, environmental improvements would eventually occur as economies grow. Consequently, humanity could, without significant deviations, go back to business as usual and still achieve environmental sustainability (Stern 2004). However, studies have observed that the relationship might be N-shaped (e.g., Bhattarai et al. 2009; Álvarez-Herranz and Balsalobre Lorente 2016), which suggests that environmental degradation will start to rise again beyond a certain income level. Yet, to our knowledge, no previous study has examined the N-shaped relationship between CO_2_ emissions and GDP per capita using panel quantile analysis while including additional explanatory variables, such as renewable energy consumption, technological development, trade, and institutional quality.

The aim of this study is to evaluate the N-shaped EKC. To this end, we analyze how different countries’ environmental degradation is affected by their economic development. Further, we compare three different groups of countries: lower-middle-income countries, upper-middle-income countries, and high-income countries. There are several economic reasons for categorizing countries into different income groups. For example, it is important to study middle-income countries separately, since these countries are home to 73% of the world’s poorest people and five billion out of the world’s seven billion people live there. Further, middle-income countries are the major drivers of the global growth (World Bank 2017a). Middle-income countries are a diverse group of countries ranging from small nations to major engines in global growth. We therefore break down middle-income economies in two groups, divided by their income, to control for their diverse nature and the different challenges they might face. Since middle-income countries are not as developed as high-income countries, they do not extend as far on the EKC. In order to analyze a wider range of the EKC, we therefore include high-income countries as a benchmark.

Since environmental degradation is not only affected by economic development, we also include variables to control for the effects of renewable energy consumption, technological development, trade, and institutional quality on environmental degradation. We aim to answer the following research questions: What does the relationship between environmental degradation and economic development look like for lower-middle-income countries, upper-middle-income countries, and high-income countries? How can environmental degradation be explained by renewable energy use, technological development, trade, and institutional quality?

We utilized panel quantile analysis in order to address our research questions. We chose to mainly focus on the quantile panel regressions as it provides a more comprehensive picture of the relationship between the variables in comparison with pooled OLS and fixed effects models. Annual data were obtained from the World Development Indicators (WDI) and from the Freedom House database, covering 74 countries over the period of 1994–2012. This was the longest and most up-to-date time series available without reducing our sample, due to missing data. We estimated regression models both for the total sample and the three income groups separately.

This paper contributes to the existing literature by improving our knowledge of the possible N-shaped relationship between income and environmental degradation. The existing literature has mainly focused on different regions, on OECD countries, or on larger samples of countries. Although a small number of studies have focused on different income groups, none of them have to our knowledge used panel quantile regressions. Therefore, there is a gap in the existing EKC literature, which we intend to fill by combining the use of quantile regressions with income classifications.

## Literature review

According to the EKC, first proposed by Grossman and Krueger (1991), the relationship between economic growth and environmental degradation has the shape of an inverted U. The N-shaped EKC suggests that the original EKC hypothesis will not hold in the long run. Instead, beyond a certain income level, increased income might once again lead to a positive relationship between economic growth and environmental degradation (de Bruyn et al. 1998). Torras and Boyce (1998) suggest that the N-shaped relationship occurs when the scale effect overcomes the composition and technical effects. This might be the consequence of reduced possibilities to further improve distribution of industries or because of diminishing returns on technological changes (Torras and Boyce 1998; Álvarez-Herranz and Balsalobre Lorente 2015, 2016).

There are several reviews that covers the existing literature on the EKC (Dinda 2004; Stern 2004; Culas 2012; Kaika and Zervas 2013). The inverted U-shaped relationship between income and environmental degradation has been confirmed by several researchers. For example, when using a fixed effects model (FEM), Leitão (2010) finds it for 94 countries with different development levels and Culas (2012) finds it for 23 African countries. Culas (2012) also finds the inverted U-shaped EKC for 9 Latin American countries when using a random effects model (REM). This shape has also been found for 29 OECD countries when using a stochastic impacts by regression on population, affluence, and technology model (Shafiei and Salim 2014) and for 24 European countries when using a pooled mean group approach (Ahmed et al. 2016). Further, Al-Mulali et al. (2016) find the inverted U-shaped relationship for Europe, East Asia and the Pacific, South Asia, and the Americas when using dynamic OLS. It is also found for various countries, when using quantile regressions with fixed effects (You et al. 2015). When using quantile regressions, the inverted U-shaped EKC is found for ASEAN-5 (Duan et al. 2016) and for 19 APEC countries.^1^ However, some of the studies finding an inverted U-shaped EKC have not included the cubic form of income. These studies are thereby ignoring the possibility of an N-shaped EKC (e.g., Culas 2012; Duan et al. 2016; Zhang et al. 2016). Lee et al. (2009) demonstrate this by finding an inverted U-shaped EKC when using a quadratic model and an N-shaped EKC when using a cubic model.

Even though the N-shaped EKC is considered to be a new phenomenon, it was found as early as in the 1990s. Grossman and Krueger (1995) and Panayotou (1997) find an N-shaped relationship between economic development and sulfur dioxide (SO_2_). In both cases, few observations existed after the second turning point, as it was in the extreme end of the data set, and the N-shape was therefore dismissed. Moomaw and Unruh (1997) find the N-shaped EKC when using FEM and cross-sectional OLS. However, the authors also used a structural transition model which indicated that the shift to declining CO_2_ emissions most likely was a result of the 1973 oil crisis. The N-shaped EKC is also found for Austria when using pooled OLS (Friedl and Getzner 2003) and for 28 OECD countries when using generalized least squares (Álvarez et al. 2015). When using FEM, the N-shaped relationship is found for 15 Latin American countries (Bhattarai et al. 2009), 28 OECD countries (Álvarez-Herranz and Balsalobre Lorente 2015), and 17 OECD countries (Álvarez-Herranz and Balsalobre Lorente 2016).

The inverted U-shaped EKC and the N-shaped EKC has also been found by the same researchers but for different regions or environmental degradation measures. For example, when using REM Grossman and Krueger (1995) find the N-shaped EKC for SO_2_, but the inverted U-shaped relationship for other environmental indicators. Further, López-Menéndez et al. (2014) find the inverted U-shaped EKC for EU27 countries where at least 20% of the country’s electricity is generated from renewable energy sources. However, an N-shaped relationship is found for the EU27 countries where less than 20% of the country’s electricity is generated from renewable energy sources.

In recent years, the impact of renewable energy on environmental degradation has been widely studied. Various studies indicate that greenhouse gas (GHG) emissions can be reduced as fossil fuels are replaced with renewable energy (López-Menéndez et al. 2014; Shafiei and Salim 2014; Álvarez-Herranz and Balsalobre Lorente 2015, 2016; Al-Mulali et al. 2016). Thereby, renewable energy consumption should have a negative impact on environmental degradation.

Recently, Shahbaz et al. (2017) showed that utilization of energy efficiency is important for sustainable economic development in the long run, for 25 developed economies during the period of 1970–2014. Attiaoui et al. (2017) found a unidirectional causality from renewable energy consumption to output for 22 African countries during the period of 1990–2011. Lu (2017) finds that a long run equilibrium exists among renewable energy consumption, carbon emission, and GDP using panel data for 24 Asian countries during the period of 1990–2012. Paramati et al. (2017) analysis on Next 11 countries suggests that renewable energy production and various economic activities are required for sustainable economic development.

We consider the several control variables in our empirical investigation. In line with the previous literature, we identify particularly three important variables: technology and innovation, trade or openness, and institutional quality. All, strongly connected with environmental policies. For instance, several studies use research and development and patent to measure countries’ technology and innovation (Álvarez et al. 2015; Álvarez-Herranz and Balsalobre Lorente 2015, 2016; Ahmed et al. 2016). They found that technological innovation has a negative effect on environmental degradation. Further, the empirical evidence on the relationship between trade and environmental degradation is inconclusive.^2^ Moreover, some studies have suggested that institutional variables such as corruption and level of democracy might be important determinants of environmental policies (Zhang et al. 2016; Leitão 2010; Panayotou 1997; Torras and Boyce 1998).

## Data and preliminary analysis

We include three groups of countries in the sample: high-income countries, upper-middle-income countries, and lower-middle-income countries. These classifications are defined in accordance with the World Bank (2017b). We choose not to include low-income economies in the study, because these countries’ contribution to the global share of GDP as well as to CO_2_ emissions is minimal. It would also be problematic to find balanced data for the low-income countries. In contrast, middle-income countries have had a rising importance for the global economy with an increasing industrial output and, hence, rising emissions. Since middle-income economies are expected to grow even more, it is important to investigate how this will affect the global environment. By using high-income economies as a benchmark, we can compare these groups of countries to get a better understanding of what we need to do in order to achieve sustainable development.

This study is based on annual data for CO_2_ emissions per capita, real GDP per capita, renewable energy, technological development, trade, and institutional quality. Data for institutional quality are obtained from the Freedom House (2017a) database and remaining series are downloaded from the WDI, obtained from the World Bank (2017c). The dataset covers an unbalanced panel of 74 countries or a balanced panel of 55 countries over the time period 1994 to 2012. Since we use lags of 1 year for technological development, the corresponding time period for this variable is 1993 to 2011. We include all lower-middle-income countries, upper-middle-income countries, and high-income countries with available data for the selected variables over the time period. The included countries are shown in Table 11 (see Appendix 1).

We use CO_2_ emissions (*CO*_*2*_) as a proxy for environmental degradation, as is common in this field of research (Álvarez et al. 2015; Álvarez-Herranz and Balsalobre Lorente 2016). Further, CO_2_ emissions represent more than 80% of the total global GHG emissions (World Bank 2014). The variable does not measure CO_2_ emissions from imported goods and do not subtract emissions from exported goods. Thus, using this variable leads to a production-based approach of the EKC. The CO_2_ series is measured in metric tons per capita which enables us to adjust for the effect of population growth on the pollution level. To measure the effect of economic growth on environmental degradation, we use real GDP per capita (*GDP*). Substitution to greener energy sources might decrease environmental degradation. As a measure for this substitution effect, we use renewable energy consumption as the share of total energy consumption (*REN*). To measure the technological development of a country, we use patent applications (*R&D*) as a proxy. We combine two different series, one for patents applied by residents and one for those applied by nonresidents. We use an aggregate measure of patents in order to capture the total effect of a country’s technological development on the environment. Another possible variable for measuring technological development would be research and development expenditure as a share of GDP. According to Popp (2012), the collection of data for expenditures in research and development can differ between countries and this data is therefore noisy. The available data is also limited for this variable and patent applications is a commonly used proxy for technology (Ahmed et al. 2016). To measure the effects of trade on environmental degradation, we use trade as share of GDP (*TRD)* as a proxy. The variable is constructed as the sum of exports and imports of goods and services measured as the share of GDP. All data are extracted from the World Development Indicator, WDI (World Bank 2017c). As a proxy for the institutional quality in a country, we use the Freedom House (2017a) political rights index and the civil liberties index (*INS*).^3^

### Descriptive statistics

Table 1 presents the descriptive statistics of the dependent and the explanatory variables for the total sample of 74 countries over a period of 19 years. In order to minimize the issue of heteroscedasticity and to improve the comparability with previous studies, all variables except for *INS* are expressed in natural logarithms, since *INS* is an index ranging from 1 to 13. Also, when using the natural logarithm on *INS*, it gets further away from a normal distribution with skewness close to − 2 and a high value for the kurtosis.

**Table 1:** Descriptive statistics for total sample

Variable	Mean	Median	Max	Min	Std. dev.	Skewness	Kurtosis	*N*
CO_2_	1.42	1.74	3.23	− 1.97	1.08	− 1.00	3.42	1406
GDP	9.08	9.09	11.61	5.90	1.36	− 0.22	2.05	1406
REN	2.38	2.67	4.53	− 4.80	1.51	− 1.53	7.07	1406
R&D	7.48	7.43	13.17	1.61	2.04	0.39	3.17	1406
TRD	4.26	4.24	6.09	2.75	0.54	0.24	3.75	1406
INS	9.43	11.00	13.00	1.00	3.72	− 0.78	2.32	1406

All variables except for *INS* are expressed in natural logarithms in this table, and the following tables. All variables expect *INS* are obtained from WDI (World Bank 2017c). The indexes used in *INS* are obtained from Freedom House (2017a)

*CO*_*2*_ CO_2_ emissions measured in metric tons per capita, *GDP* GDP per capita measured in constant 2010 US dollar, *REN* renewable energy consumption as a share of total energy consumption, *R&D* patent application from residents and nonresidents, *TRD* the sum of exports and imports as share of GDP, *INS* the sum of a political rights index and a civil liberties index minus 15

As we can see in Table 1, we have some excessive skewness to the left for *REN* and *CO*_*2*_, however a range of ± 2 from a normal distribution with skewness of 0 can be seen as acceptable. Further, we see some excessive kurtosis of 7.07 for *REN* in comparison to a normal distribution with a kurtosis of 3. However, the other variables do not express any excessive deviations from a normal distribution.

The correlations between all variables are shown in Table 2. The value of all correlations between the explanatory variables are way below 0.7, which we use as a rule of thumb for stronger correlation. However, the correlation between *GDP* and *INS* is 0.69, which might lead to problems with multicollinearity when the variables are estimated in the same model. Nevertheless, excluding one of the variables might lead to omitted variable bias. Regarding the rest of the variables, we do not consider their correlations to be of any concern.

**Table 2:** Pearson correlations

	CO_2_	GDP	REN	R&D	TRD	INS
CO_2_	1.00	–	–	–	–	–
GDP	0.80	1.00	–	–	–	–
REN	− 0.56	− 0.26	1.00	–	–	–
R&D	0.46	0.38	− 0.22	1.00	–	–
TRD	0.25	0.18	− 0.21	− 0.32	1.00	–
INS	0.43	0.69	0.19	0.18	0.05	1.00

## Methodology and hypotheses

### Model

The theoretical relationship between environmental degradation and economic growth is usually described as follows (Grossman and Krueger 1991; Stern 2004):

1 $$ {\mathrm{GHG}}_{it}={\alpha}_{it}+{\beta}_1{\mathrm{GDPpc}}_{it}+{\beta}_2{\mathrm{GDPpc}}_{it}^2+{\beta}_3{\mathrm{GDPpc}}_{it}^3+{\beta}_4{Z}_{it}+{\varepsilon}_{it}, $$

where GHG refers to the greenhouse gas emissions, that is, environmental degradation, GDPpc stands for income per capita, and *Z* contains all other variables that might affect environmental quality. The coefficient *α*_*it*_ measures the average environmental pressure when income has no influence, *β* refers to the direction and importance of the exogenous variables, and *ε*_*it*_ is the error term. Depending on the sign of the different *β* parameters related to income, the EKC will adopt different shapes (Álvarez-Herranz and Balsalobre Lorente 2016):

(i) If β_1_ = β_2_ = β_3_ =0, there will be either a flat pattern or no relationship between environmental degradation and income.
(ii) If β_1_ >0 and β_2_ = β_3_ = 0, there will be a monotonic increasing relationship such that environmental degradation increases along with economic growth.
(iii) If β_1_ < 0 and β_2_ = β_3_ = 0, there will be a monotonic decreasing relationship between environmental deterioration and income.
(iv) If β_1_ > 0 and β_2_ < 0 and β_3_ = 0, we will see the classical inverted U-shaped EKC.
(v) If β_1_ < 0 and β_2_ > 0 and β_3_ = 0, there will be a U-shaped relationship between environmental degradation and income.
(vi) If β_1_ > 0 and β_2_ < 0 and β_3_ > 0, there will be a cubic polynomial or N-shaped relationship between environmental deterioration and income.
(vii) If β_1_ < 0 and β_2_ > 0 and β_3_ < 0, there will be an inverted, or opposite, N-shaped relationship between environmental degradation and economic growth.

We estimated an empirical model consisting of a relationship between CO_2_ emissions (*CO*_*2*_) and the following explanatory variables: income (*GDP*), renewable energy consumption (*REN*), technological development (*R&D*), trade (*TRD*), and institutional quality (*INS*). The model is given by

2 $$ {\displaystyle \begin{array}{l}{\mathrm{CO}}_{2 it}=\alpha +{\beta}_1{\mathrm{GDP}}_{it}+{\beta}_2{{\mathrm{GDP}}^2}_{it}+{\beta}_3{{\mathrm{GDP}}^3}_{it}+{\beta}_4{\mathrm{REN}}_{it}\\ {}\kern3em +{\beta}_5R\&{D}_{i\left(t-1\right)}+{\beta}_6{\mathrm{TRD}}_{it}+{\beta}_7{\mathrm{INS}}_{it}+{\varepsilon}_{it}\end{array}} $$

where *i* and *t* are indexes for country and time. All variables except for *INS* are expressed in natural logarithms. We assume that there is some delay before innovations are implemented in a society. In accordance with previous literature (e.g., Álvarez et al. 2015; Álvarez-Herranz and Balsalobre Lorente 2015) we, therefore, choose to lag *R&D*. Popp (2012) argues that patents not only measure the coming years’ innovative output, but also measure the level of innovative activity in the country today. As we want *R&D* to reflect both the innovative activity level and innovative output in a country, we choose to lag *R&D* by 1 year. Further, increasing the lag length would not be possible without reducing our sample or imputing a lot of units, due to missing data for the variable for years earlier than 1993.

### Quantile regression

The statistical distribution of data often has an unequal variation and the relationship between the variables can therefore change between the locations on the dependent variable’s conditional distribution. Estimations based on the mean values, such as pooled OLS, FEM, and REM, can therefore give incorrect results (Cade and Noon 2003). Quantile regressions evaluate the different points on the conditional distribution of the dependent variable and can thereby provide a more complete picture of the relationship between the variables (Cade and Noon 2003). The motivation for panel quantile approach is to capture the heterogeneous structure of the different income groups and different market condition, as the pooled OLS only consider the mean. We therefore chose to complement the pooled OLS and FEM with a quantile regression analysis.

In quantile regressions, the conditional distribution of the dependent variable is divided into different quantiles, where the 50th quantile represent the median (Hübler 2017). Therefore, quantile regressions are more robust to outliers than estimation techniques referring to the mean. Hübler (2017) also states that the differences between the median and the mean can be large for variables such as CO_2_ and GDP. Thus, quantile regression is an interesting approach to the N-shaped EKC hypothesis, because of the possibilities to estimate different slopes for different quantiles. Given *x*_*i*_, the conditional quantile of *y*_*i*_ is expressed as

7 $$ {Q}_{y_{it}}\left(\boldsymbol{\tau} |{\boldsymbol{x}}_{it}\right)={\boldsymbol{x}}_{it}^{\tau }{\boldsymbol{\beta}}_{\tau } $$

where ${Q}_{y_{it}}\left(\tau |{x}_{it}\right)$ means the *τ*th quantile of the dependent variable, ${\boldsymbol{x}}_{it}^{\tau }$ is the vector of explanatory variables for each country *i* at year *t* for quantile *τ*, and ***β***_*τ*_ symbolizes the slopes of the explanatory variable for quantile *τ* (Duan et al. 2016). To test the robustness of our variables, we estimated regressions on a balanced dataset and regressions where we excluded renewable energy consumption. We decided to estimate a model which only included balanced data to control for our imputed units. Further, we chose to estimate a model where we excluded renewable energy consumption, since the variable indirectly could measure technological development in the field of renewable energy.

### Hypotheses

In accordance with the economic theories and empirical evidence presented earlier in the paper, we formulated hypotheses regarding the directions of the β-parameters. Table 3 shows the expected effect of each explanatory variable on *CO*_*2*_.

**Table 3:** Hypotheses

Explanatory variable	Effect on CO_2_ emissions per capita
GDP	+
GDP^2^	−
GDP^3^	+
REN	−
R&D	−
TRD	+ for middle-income countries, − for high-income countries
INS	−

In accordance with the theory of the N-shaped EKC, we hypothesized *GDP* to have a positive effect on CO_2_ emissions, reflecting the increasing emissions in the early stages of growth. *GDP* ^*2*^ should show a negative effect indicating decreasing emissions beyond the first turning point, while *GDP* ^*3*^ should show a positive sign, as emissions once again increase with income. We hypothesized that a higher share of renewable energy sources will reduce CO_2_ emissions, indicating a negative sign of renewable energy. More efficient technology or emission specific changes in processes should reduce emissions and therefore we hypothesized technological development to have a negative effect on CO_2_ emissions. In accordance with the pollution haven hypothesis, we hypothesized that trade will lead to increasing emissions for the middle-income countries, especially for the lower-middle-income countries, and decreasing emissions for high-income countries. Finally, we hypothesized institutional quality to have a negative effect on CO_2_ emissions, as institutions should be important components for reducing emissions.

## Results and discussions

### Preliminary checkups

According to the VIF test, presented in Table 14 (see Appendix 2), no multicollinearity exists in our model. All VIF values are below 5, with the highest value of 3.123, indicating that there is no problem with multicollinearity. The results from the panel data unit root tests are presented in Table 4. The table shows the results from the Fisher PP-statistics (Maddala and Wu 1999) and the LLC-statistics ( Levin et al. 2002). All tests were estimated both with a constant and a trend. Rejection of the null hypothesis indicates that the series are stationary.

**Table 4:** Panel data unit root tests

	Level	
Variable	Fisher PP-statistic	LLC-statistic
CO_2_	177.621**	− 0.718
GDP	77.757	− 17.182***
REN	231.783***	− 4.684***
R&D	258.250***	− 9.618***
TRD	197.093***	− 7.029***
INS	177.976***	− 4.942***

***, **, and * indicate significant *p* values at the 1, 5, and 10% level, respectively. Both a constant and a trend were used in the tests

The tests show that all series are I(0) stationary. However, as can be seen in the table, only the PP-statistics rejects the null hypothesis for the *CO*_*2*_ series, while only the LLC-statistics rejects the null hypothesis for the *GDP* series. Since we perform these tests to check the statistical properties of the series, rather than deciding between using the variables in level or first difference, the different results between the PP- and LLC-statistics for *CO*_*2*_ and *GDP* are of less importance. We proceeded by estimating the pooled OLS, FEM, and the quantile regressions in level.

### Pooled OLS, fixed effects model, and quantile model

The results from the pooled OLS estimations and the FEM estimations for the unbalanced panels are presented in Table 5. The FEM estimations are fixed both over the individuals and the time period. We also present the *p* values from the Hausman tests in the table. Estimations (1) and (5) show the results for the total sample, estimations (2) and (6) cover the lower-middle-income countries, estimations (3) and (7) show the results for the upper-middle-income countries, and estimations (4) and (8) cover the high-income countries. The results from the quantile regressions for the total sample and for the different classifications are presented in Tables 6, 7, 8, and 9. Table 10 summarizes the results from all quantile regressions. Table 10 shows that the quantile regression results regarding the relationship between income and environmental degradation are inconclusive. The N-shaped EKC is found in half of the regressions, but some results also indicate that the relationship might have the shape of an inverted N.

**Table 5:** Results from pooled OLS and FEM estimations

	Pooled OLS estimator				Fixed effects model			
Explanatory variables	(1) Total sample	(2) LMIC	(3) UMIC	(4) HIC	(5) Total sample	(6) LMIC	(7) UMIC	(8) HIC
GDP	4.319*** (1.159)	31.136*** (11.025)	− 15.357 (21.846)	17.924* (10.162)	− 0.014 (1.116)	9.872 (16.649)	1.903 (10.655)	− 15.913** (7.720)
GDP^2^	− 0.361*** (0.134)	− 4.457*** (1.551)	2.005 (2.664)	− 1.737* (1.008)	0.109 (0.131)	− 1.394 (2.360)	− 0.149 (1.270)	1.666** (0.780)
GDP^3^	0.011** (0,005)	0.214*** (0.072)	− 0.085 (0.108)	0.057* (0.033)	− 0.006 (0.005)	0.069 (0.111)	0.005 (0.050)	− 0.057** (0.026)
REN	− 0.230*** (0.011)	− 0.533*** (0.014)	− 0.278*** (0.023)	− 0.172*** (0.014)	− 0.257*** (0.013)	− 0.529*** (0.074)	− 0.229*** (0.022)	− 0.185*** (0.016)
R&D	0.102*** (0.008)	0.186*** (0.010)	0.143*** (0.016)	0.027*** (0.009)	0.068*** (0.009)	0.120*** (0.037)	0.082*** (0.012)	0.004 (0.013)
TRD	0.284*** (0.027)	0.236*** (0.041)	0.522*** (0.044)	0.046 (0.032)	0.116*** (0.022)	0.135 (0.091)	0.123*** (0.036)	0.084** (0.039)
INS	0.019*** (0.006)	− 0.010* (0.006)	0.023** (0.010)	0.042*** (0.009)	0.005 (0.004)	0.002 (0.013)	0.008** (0.004)	0.000 (0.009)
Intercept	− 17.444	− 73.461	36.260	− 61.033	− 3.101	− 24.385	− 7.956	51.741
Hausman	–	–	–	–	0.342	0.827	0.617	0.372
Observations	1406	323	380	703	1406	323	380	703
Countries	74	17	20	37	74	17	20	37
*R* ^2^	0.816	0.920	0.617	0.504	0.989	0.987	0.980	0.960
Adjusted *R* ^2^	0.815	0.919	0.610	0.499	0.988	0.985	0.978	0.956

*LMIC* lower-middle-income countries, *UMIC* upper-middle-income countries, *HIC* high-income countries

***, **, and * indicate significant *p* values at the 1, 5, and 10 % level, respectively. Standard errors are presented in the parentheses

**Table 6:** Results from quantile regression for the total sample

Explanatory variables	10th	20th	30th	40th	50^th^	60th	70th	80^th^	90th	95th
GDP	− 1.790 (1.667)	− 6.581*** (1.614)	− 4.043*** (1.400)	1.052 (1.743)	5.545*** (1.148)	4.945*** (1.478)	4.109*** (1.114)	6.470*** (1.551)	4.223 (3.485)	0.158 (0.486)
GDP^2^	0.385** (0.185)	0.940*** (0.179)	0.641*** (0.163)	0.047 (0.205)	− 0.500*** (0.135)	− 0.461*** (0.167)	− 0.373*** (0.130)	− 0.623*** (0.173)	− 0.356 (0.374)	0.067 (0.056)
GDP^3^	− 0.019*** (0.007)	− 0.040*** (0.007)	− 0.028*** (0.006)	− 0.006 (0.008)	0.016*** (0.005)	0.015** (0.006)	0.012** (0.005)	0.021*** (0.006)	0.011 (0.013)	− 0.004** (0.002)
REN	− 0.206*** (0.009)	− 0.158*** (0.012)	− 0.148*** (0.010)	− 0.154*** (0.011)	− 0.185*** (0.017)	− 0.270*** (0.017)	− 0.297*** (0.018)	− 0.293*** (0.009)	− 0.256*** (0.015)	− 0.253*** (0.006)
R&D	0.068*** (0.006)	0.073*** (0.009)	0.087*** (0.007)	0.089*** (0.008)	0.099*** (0.009)	0.116*** (0.008)	0.110*** (0.010)	0.107*** (0.008)	0.081*** (0.013)	0.069*** (0.006)
TRD	0.302*** (0.022)	0.368*** (0.035)	0.355*** (0.017)	0.314*** (0.024)	0.345*** (0.023)	0.308*** (0.030)	0.242*** (0.038)	0.233*** (0.030)	0.219*** (0.040)	0.269*** (0.016)
INS	0.022*** (0.007)	− 0.005 (0.008)	− 0.011** (0.005)	− 0.004 (0.006)	0.006 (0.004)	0.027*** (0.005)	0.029*** (0.008)	0.017*** (0.006)	0.021* (0.012)	0.043*** (0.003)
Intercept	− 1.931	11.565	4.615	− 9.356	− 21.387	− 18.532	− 15.423	− 22.427	− 15.802	− 2.775

***, **, and * indicate significant *p* values at the 1, 5, and 10% level, respectively. The standard errors, presented in the parentheses, are obtained with a bootstrap of 500

**Table 7:** Results from quantile regression for lower-middle-income countries

Explanatory variables	10th	20th	30th	40th	50th	60th	70th	80th	90th	95th
GDP	40.733* (22.708)	37.528** (18.940)	33.957** (14.890)	31.983*** (12.110)	31.701** (13.582)	9.085 (19.349)	15.229 (17.187)	11.520 (13.825)	25.203 (18.766)	17.236 (21.340)
GDP^2^	− 5.796* (3.124)	− 5.305** (2.611)	− 4.796** (2.063)	− 4.498*** (1.692)	− 4.485** (1.906)	− 1.451 (2.737)	− 2.109 (2.392)	− 1.585 (1.923)	− 3.553 (2.668)	− 2.512 (3.052)
GDP^3^	0.276* (0.143)	0.251** (0.119)	0.227** (0.095)	0.212*** (0.078)	0.213** (0.089)	0.078 (0.129)	0.099 (0.111)	0.074 (0.089)	0.168 (0.126)	0.123 (0.145)
REN	− 0.524*** (0.025)	− 0.505*** (0.029)	− 0.481*** (0.022)	− 0.484*** (0.015)	− 0.493*** (0.015)	− 0.481*** (0.018)	− 0.514*** (0.022)	− 0.533*** (0.019)	− 0.525*** (0.019)	− 0.608*** (0.092)
R&D	0.201*** (0.021)	0.209*** (0.022)	0.236*** (0.012)	0.229*** (0.013)	0.225*** (0.014)	0.217*** (0.018)	0.162*** (0.017)	0.159*** (0.017)	0.159*** (0.020)	0.157*** (0.018)
TRD	0.256*** (0.071)	0.273*** (0.068)	0.241*** (0.061)	0.234*** (0.058)	0.199*** (0.063)	0.069 (0.064)	0.149** (0.062)	0.197*** (0.040)	0.227*** (0.038)	0.194*** (0.072)
INS	− 0.042*** (0.007)	− 0.038*** (0.007)	− 0.036*** (0.007)	− 0.042*** (0.007)	− 0.045*** (0.008)	− 0.039*** (0.012)	− 0.005 (0.014)	0.011* (0.007)	0.019*** (0.006)	0.015 (0.016)
Intercept	− 96.551	− 89.853	− 81.654	− 77.106	− 75.730	− 19.260	− 37.220	− 28.480	− 60.130	− 39.387

***, **, and * indicate significant *p* values at the 1, 5, and 10% level, respectively. The standard errors, presented in the parentheses, are obtained with a bootstrap of 500

**Table 8:** Results from quantile regression for upper-middle-income countries

Explanatory variables	10th	20th	30th	40th	50th	60th	70th	80th	90th	95th
GDP	43.444* (24.478)	13.616 (24.351)	9.607 (26.289)	− 16.357 (34.620)	− 31.844 (28.634)	− 74.154** (30.883)	− 92.891** (35.934)	− 59.712* (34.981)	33.700 (37.743)	4.165 (44.630)
GDP^2^	− 5.101* (2.999)	− 1.531 (2.988)	− 1.035 (3.219)	1.974 (4.202)	3.930 (3.446)	8.987** (3.685)	11.238*** (4.287)	7.137* (4.218)	− 4.201 (4.568)	− 0.395 (5.695)
GDP^3^	0.201 (0.122)	0.059 (0.122)	0.039 (0.131)	− 0.077 (0.169)	− 0.159 (0.138)	− 0.360** (0.146)	− 0.451*** (0.170)	− 0.282* (0.169)	0.174 (0.183)	0.013 (0.240)
REN	− 0.158*** (0.028)	− 0.241*** (0.040)	− 0.288*** (0.036)	− 0.345*** (0.035)	− 0.396*** (0.032)	− 0.377*** (0.033)	− 0.386*** (0.035)	− 0.364*** (0.038)	− 0.323*** (0.045)	− 0.315*** (0.099)
R&D	0.151*** (0.052)	0.166*** (0.030)	0.145*** (0.024)	0.133*** (0.044)	0.178*** (0.017)	0.168*** (0.016)	0.177*** (0.015)	0.168*** (0.016)	0.157*** (0.017)	0.078 (0.098)
TRD	0.543*** (0.049)	0.468*** (0.045)	0.468*** (0.039)	0.509*** (0.056)	0.531*** (0.061)	0.560*** (0.060)	0.566*** (0.056)	0.603*** (0.062)	0.689*** (0.112)	0.222 (0.725)
INS	− 0.022 (0.024)	− 0.013 0.019)	− 0.022 (0.016)	− 0.003 (0.018)	0.015 (0.012)	0.027*** (0.009)	0.038*** (0.008)	0.045*** (0.011)	0.083*** (0.013)	0.052 (0.053)
Intercept	− 126.169	− 42.745	− 31.635	42.640	83.154	200.904	252.829	163.612	− 91.701	− 13.728

***, **, and * indicate significant *p* values at the 1, 5, and 10% level, respectively. The standard errors, presented in the parentheses, are obtained with a bootstrap of 500

**Table 9:** Results from quantile regression for high-income countries

Explanatory variables	10th	20th	30th	40th	50th	60th	70th	80th	90th	95th
GDP	99.187** (44.082)	− 7.424 (47.173)	38.070* (19.701)	31.216* (16.572)	15.636 (15.596)	32.518** (14.150)	46.389*** (8.835)	7.975 (35.735)	1.845 (40.724)	− 0.776 (50.320)
GDP^2^	− 9.337** (4.360)	0.956 (4.775)	− 3.698* (1.935)	− 3.022* (1.624)	-1.486 (1.538)	− 3.206** (1.413)	− 4.692*** (0.883)	− 0.863 (3.585)	− 0.222 (4.090)	0.095 (4.848)
GDP^3^	0.293** (0.143)	− 0.038 (0.161)	0.121* (0.063)	0.098* (0.053)	0.048 (0.050)	0.106** (0.047)	0.159*** (0.029)	0.032 (0.119)	0.010 (0.136)	− 0.003 (0.155)
REN	− 0.220*** (0.021)	− 0.187*** (0.025)	− 0.166*** (0.020)	− 0.151*** (0.014)	− 0.136*** (0.014)	− 0.123*** (0.016)	− 0.121*** (0.021)	− 0.168** (0.073)	− 0.152** (0.076)	− 0.186*** (0.049)
R&D	− 0.030 (0.019)	0.004 (0.016)	0.011 (0.011)	0.010 (0.008)	0.014* (0.008)	0.007 (0.008)	0.036** (0.019)	0.064*** (0.017)	0.075*** (0.016)	0.059** (0.026)
TRD	0.029 (0.031)	0.040 (0.079)	0.016 (0.046)	0.027 (0.035)	0.020 (0.037)	0.020 (0.035)	− 0.041 (0.063)	0.023 (0.070)	0.135 (0.083)	0.193* (0.101)
INS	0.068*** (0.011)	0.046** (0.021)	0.019 (0.013)	0.014 (0.009)	0.009 (0.009)	0.008 (0.010)	0.007 (0.013)	0.039 (0.045)	0.046 (0.046)	0.087 (0.033)
Intercept	− 349.324	17.970	− 129.645	− 106.494	− 53.791	− 108.620	− 151.146	− 23.937	− 4.682	2.350

***, **, and * indicate significant *p* values at the 1, 5, and 10% level, respectively. The standard errors, presented in the parentheses, are obtained with a bootstrap of 500

**Table 10:** Summary of the quantile regression estimations

	Total sample			LMIC			UMIC			HIC		
Quantile	L	M	H	L	M	H	L	M	H	L	M	H
GDP	−	+	+	+	+	/	/	/	−	+	+	/
GDP^2^	+	−	−	−	−	/	/	/	+	−	−	/
GDP^3^	−	+	+	+	+	/	/	/	−	+	+	/
REN	−	−	−	−	−	−	−	−	−	−	−	−
R&D	+	+	+	+	+	+	+	+	+	/	/	+
TRD	+	+	+	+	+	+	+	+	+	/	/	/
INS	/	/	+	−	−	+	/	/	+	+	/	/

+ means that the variable has a significant positive effect on CO_2_ emission for at least two out of three quantiles, − means that that the variable has a significant negative effect on CO_2_ emission for at least two out of three quantiles, and / means that no significant or uniform effect could be found

*L* lower quantiles, the 10th, 20th, and 30th quantile; *M* middle quantiles, 40th, 50th, and 60th quantiles; *H* for higher quantiles, 70th, 80th, 90th, and 95th quantiles

### Discussion

According to our hypothesis and the theoretical framework, an N-shaped relationship between income and environmental degradation should be expected in the estimations. However, as seen in Tables 5, 6, 7, 8, 9, and 10, the results are inconclusive both between classifications and between the different methods used. The pooled OLS estimations confirm our hypothesis of an N-shaped EKC for the total sample, lower-middle-income countries, and high-income countries. However, when estimating the regressions with FEM, no N-shaped relationship is found for any of the classifications. Instead, the high-income countries show an inverted N-shaped relationship. This is in contrast to the results of Álvarez-Herranz and Balsalobre Lorente (2015, 2016) where estimations with FEM generates the expected N-shaped EKC. Even though the pooled OLS is chosen as the main method in this paper according to the Hausman tests, the results from estimations with FEM should still be consistent and need to be analyzed. Since the N-shaped curve is found when using the pooled OLS estimator, but not when using FEM, it is possible that the heterogeneity eliminates the N-shaped EKC. Observable individual specific effects that are constant over time cannot be separated from non-observable individual specific effects when using FEM. Some effects of *GDP* might therefore be captured in the individual intercept, eliminating the N-shaped EKC. For example, being a rich and highly educated country, which should be correlated to *GDP*, might be included in the individual intercept if this is a factor that is constant over time.

The results from the quantile regressions are also inconclusive. None of Tables 6, 7, 8, 9, and 10 show uniform results for any of the classifications regarding the relationship between income and environmental degradation. Even though the pooled OLS showed an N-shaped EKC for the total sample, lower-middle-income countries, and high-income countries, only some of the quantiles confirm these results. These inconclusive results might depend on heterogeneity between and within these income groups. A further breakdown of the included countries and their specific characteristics, such as environmental laws and composition of industries, might therefore be needed to fully understand why the N-shaped EKC is only apparent in some of the quantiles.

One interesting finding is that upper-middle-income countries differ from the other classifications regarding the relationship between income and CO_2_ emissions. In contrast to the other groups of countries, none of the methods generated an N-shaped EKC in any estimation for the upper-middle-income countries. In fact, some of the quantiles instead show an opposite N-shaped EKC. This indicates that economic growth initially will improve environmental quality up to a certain income level where the relationship instead will be positive before it ones again becomes negative. This is an interesting finding that is difficult to explain. Possibly, it could be a consequence of a high-energy efficiency, compensating for the increased emissions caused by the scale effect. Further, it could also be a consequence of a growing amount of foreign direct investment and multinational companies operating in these countries, leading to an inflow of technology from more developed countries. Improvements in the countries’ technological frontiers could thereby outpace the scale effect, causing a negative effect of *GDP* on *CO*_*2*_. However, in most quantiles for the upper-middle-income countries, no significant relationship is found.

The inconclusive results suggest that the EKC relationship should be studied with carefulness. It is common in the research field to only use mean regressions as method, which might generate non-representative results for many of the countries included in the sample. When using quantile regressions, we see that the relationship between income and environment widely differs between quantiles. These results are in line with those of Duan et al. (2016), You et al. (2015), and Zhang et al. (2016) where the shape of the EKC also is inconclusive when using quantile regressions. Policy implications which are only based on results from mean regressions might therefore be ineffective. Further, a large part of the existing literature on the EKC omit the cubic relationship in their estimations and thereby ignore the possibility of an N-shaped EKC. In this paper, the inverted U-shaped relationship, confirmed in several previous studies, is only found in the 10th quantile for upper-middle-income countries. Thus, this is the only regression in our study that supports the original EKC hypothesis. Omitting the cubic relationship might therefore lead us to erroneously support the inverted U-shaped EKC hypothesis.

Another possible explanation for the inconclusive results might be that the relationship between income and environmental degradation is more complex than our methodology allows us to examine. The relationship might have a functional form other than those that are possible to capture by the model applied in this paper. Therefore, the estimation methods need to be further developed to test for other more complicated relationships. For example, we do not test for a non-monotonic increasing or decreasing relationship, like a cubic function with saddle point, which could be a possible shape of the relationship.

According to the theoretical framework and our hypotheses, the share of renewable energy in total energy consumption should have a negative effect on CO_2_ emissions. This hypothesis is supported by all estimations, both with the pooled OLS estimator, FEM, and quantile regressions. The results are also robust to the sensitivity analysis and are highly significant in all income groups. The robustness of this variable shows that the substitution to renewable energy is an important aspect in reducing the environmental degradation. These results are also in line with the findings in the previous literature (López-Menéndez et al. 2014; Shafiei and Salim 2014; Álvarez-Herranz and Balsalobre Lorente 2015, 2016; Al-Mulali et al. 2016). When we excluded renewable energy in the sensitivity analysis, the N-shaped EKC was no longer apparent for the total sample and lower-middle-income countries. This suggests that increasing the share of renewable energy is crucial in order to achieve a negative relationship between income and environmental degradation in the first place. However, the share of renewable energy cannot exceed 100%, which might be the reason for the second turning point of the EKC. Environmental deterioration does not only come from the use of energy, but also from other factors such as the destruction of natural resources, for example, deforestation, as well as the industrial process. When the share of renewable energy is already filled, further increases in income might therefore lead to increased pollution levels along with the scale effect.

It was hypothesized that technological development would have a negative effect on CO_2_ emissions because of greener and more efficient technologies. However, in contrast to our hypothesis, the results show a positive effect of technological development on environmental degradation. Yet, the relationship is insignificant in most estimations for the high-income-countries. The results are inconsistent with those of Álvarez et al. (2015), Ahmed et al. (2016), and Álvarez-Herranz and Balsalobre Lorente (2015, 2016), where the effect was negative. However, their studies were conducted on OCED countries and European countries, which are more developed than parts of our sample. Further, Álvarez et al. (2015) and Álvarez-Herranz and Balsalobre Lorente (2015, 2016) used energy RD&D as a proxy instead of patents. A possible reason behind the positive effect of technological development is that our proxy includes all patents and not only patents linked to cleaner technologies. Therefore, we include technological development with all characteristics, where some lead to less pollution and some lead to more.

As stated above, the effect of technological development on CO_2_ emissions is inconclusive for the high-income countries. The insignificant results shown in several quantiles and in the pooled OLS might depend on the share of environmentally related patents in these countries. It is possible that technological development might have a negative effect on CO_2_ emissions in some of the countries if these invest more in developing greener technologies than others. This heterogeneity could be a reason behind the insignificant effect in some of the estimations. It should also be noted that the positive effect of technological development decreases as we move from the lower income groups to the higher. This can indicate that as a country develops, their share of green patents will rise and thereby decrease the positive effect on CO_2_ emissions. In the sensitivity analysis, where renewable energy was excluded, technological development still has a positive effect on CO_2_ emissions, even if the impact of the variable is slightly changed. This implies that even if renewable energy captures some of the effect of technological development on CO_2_ emissions, the change in technological development is not crucial.

According to our hypotheses trade would be positive for middle-income countries but negative for high-income countries. However, previous literature is quite inconsistent for this variable. For example, Lee et al. (2009) find evidence for the PHH, while You et al. (2015) do not find any significant results for the variable. In contrast to these studies, our results show a positive effect of trade on environmental degradation for all classifications when using pooled OLS, FEM, and quantile regressions. However, the variable is not significant for the high-income countries when using pooled OLS or in any of the quantiles. The insignificant effect indicates that trade might have both positive and negative effects on CO_2_ emissions in the high-income countries. We argue that increasing transportation, as a consequence of trade, might be one reason for the positive relationship. However, in the high-income countries, this positive effect on CO_2_ emissions might be in conflict with a negative effect. When countries engage in trade, the distribution of industries change as richer countries can move their production to countries with lower costs and thereby shift their production to the service sector. This will result in a reduction in CO_2_ emissions in high-income countries. The reduction might be large enough to compensate for the increased CO_2_ emissions coming from transportation and the scale effect. Therefore, our results neither confirm nor reject the PHH.

The results for our last variable, institutional quality, is inconclusive. Most regressions show a positive effect of institutional quality on CO_2_ emissions, in contrast to our hypothesis. However, this might be a consequence of the correlation between institutional quality and GDP being 0.69. The expected negative effect on CO_2_ emissions is only confirmed in a few estimations: in the 10th to 60th quantile and the pooled OLS for the lower-middle-income countries, and in the 30th quantile for the total sample. Thus, one conclusion that can be drawn from our results is that institutional quality is most important for the lower-middle-income countries, especially for countries with lower pollution levels. A reason might be that less-developed countries often have worse political rights and civil liberties. If a country already has well-developed political rights and civil liberties, an increase in any of these variables might not affect the country as much. Improved institutions in, for example, high-income countries might therefore not have any direct impact on the environment, unless the institution is directly connected to environmental quality. However, these findings are in contrast to the results of Zhang et al. (2016), who observed a negative effect of democracy on CO_2_ emissions in the 90th to 95th quantiles and that corruption improves environmental quality in the lower quantiles. Further, our results are also inconsistent with those of Panayotou (1997), Torras and Boyce (1998), Leitão (2010), and Al-Mulali and Ozturk (2015), which all find that institutional quality has a negative effect on environmental degradation. However, when estimating their models, they used other proxies for institutional quality, which might explain the differences in results.

One reason behind the inconclusive results for institutional quality, both within this study and in comparison to previous literature, could be that the methodology for creating the indexes changes with the ideas about political rights and civil liberties. The indexes might therefore not fully reflect the impact of a change in one country’s institutions, when the common ideas in the world change in the same direction. Further, for many countries, the index does not change over the time period. This is the case for many high-income countries which have the highest level of institutional quality, according to this index, for all the measured years. It should be added that the results regarding the effect of institutional quality on CO_2_ emissions are not robust in the sensitivity analysis. The effect is negative for the total sample and lower-middle-income countries in the estimations on the balanced panels. Further, it is negative in all estimations except for upper-middle-income countries when excluding renewable energy. It is therefore possible that the variable for renewable energy captures some of the effect of environmental connected institutions, which otherwise might be included in institutional quality.

## Conclusions and policy implications

Using a pooled OLS estimator, we find evidence for an N-shaped relationship between income per capita and CO_2_ emissions for lower-middle-income countries, high-income countries, and the total sample. These results support our hypothesis of an N-shaped EKC. However, no significant relationship is found for the upper-middle-income countries. When using quantile regressions, the N-shaped EKC is only found in some of the quantiles for lower-middle-income countries, high-income countries, and the total sample, but not in any of the quantiles for the upper-middle-income countries. Even though the majority of the statistically significant results show an N-shaped EKC, the results are heterogeneous and no strong conclusions can be drawn regarding the shape of the EKC.

The inconclusive results might be a consequence of heterogeneity across and within the income groups of countries. Further breakdowns of the countries could therefore help explain the relationship between income and environmental degradation and why it differs between the classifications. The results show that the upper-middle-income countries deviate from the other income groups and no single estimation or quantile show an N-shaped EKC. A further investigation of these countries’ characteristics would therefore be needed to understand what factors that distinguish this income group from the others.

To increase the share of renewable energy is a determining factor in reducing CO_2_ emissions. This is confirmed in all estimations for all classifications and the results are highly significant. These results indicate that it is important to encourage substitution to greener energy in order to combat climate change. In contrast to our hypothesis, the results suggest that technologic development increases CO_2_ emissions. However, we argue that this is because our variable measures all advances in technology and not only those related to environmental improvements. The quantile regressions generate inconclusive results for the high-income countries, which could be a consequence of some countries having a higher share of energy related RD&D. This could explain why the effect is statistically insignificant in several quantiles and indicates that increases in energy innovation reduces CO_2_ emissions.

According to our results, trade has a positive effect on CO_2_ emissions for all classifications and methods used, but is not significant for high-income countries. We argue that this positive effect occurs as a result of increased transportation. The insignificant effect for the high-income countries indicates that trade might both have positive and negative effects in these countries. Even though our results do not support the PHH, they neither reject it. When it comes to institutional quality, our results only show the expected negative effect on CO_2_ emissions for lower-middle-income countries in the lower quantiles. This indicates that improvements in institutional quality is most important for these countries. However, these results are not consistent with the results from our sensitivity analysis, indicating that the indexes do not fully reflect the impact of change in institutional quality. It would therefore be interesting to investigate if the results for institutional quality would be the same when using other indexes.

Based on our findings, it is clear that policies need to be designed individually for each country, depending on their income level and intensity of CO_2_ emissions. There is no policy that will fit every country, since the relationship of CO_2_ emissions with income, renewable energy, technological development, trade, and institutional quality differs with country income classifications and quantiles. Our most important policy suggestion is to implement more policies that promote the substitution to renewable energy. Policies promoting technologies with less-polluting characteristics should also be implemented; this is especially important for middle-income countries. For lower-middle-income countries, it is also important to implement policies that increase the institutional quality, in terms of political rights and civil liberties.

The inconclusive results in this study regarding the shape of the EKC suggest that further research is needed to fully understand the pollution-income relationship. The relationship might have a functional form that cannot be captured by the empirical model applied in this paper. Therefore, further research should apply models which consider other possible shapes than those normally examined in EKC-studies. It is important to further investigate the relationship between income and environmental degradation in order to combat climate change and to reach a sustainable economic development.

## Appendix 1

**Table 11:** Country classification

Lower-middle-income countries	Upper-middle-income countries	High-income countries
Armenia Bangladesh Egypt, Arab Republic Guatemala India Indonesia Kenya Mongolia Pakistan Philippines Sri Lanka Tajikistan Tunisia Ukraine Uzbekistan Vietnam Zambia	Algeria Argentina Belarus Brazil Bulgaria China Colombia Ecuador Georgia Iran, Islamic Republic Kazakhstan Macedonia, FYR Malaysia Mexico Peru Romania Russian Federation South Africa Thailand Turkey	Australia Austria Belgium Canada Chile Croatia Czech Republic Denmark Estonia Finland France Germany Greece Hungary Iceland Ireland Israel Japan Korea, Republic Latvia Lithuania Luxembourg Netherlands New Zealand Norway Poland Portugal Saudi Arabia Singapore Slovak Republic Slovenia Spain Sweden Switzerland UK USA Uruguay

## Appendix 2

**Table 12:** Balanced data pooled OLS

Explanatory variables	Total sample	Lower MIC	Upper MIC	HIC
GDP	5.745*** (1.345)	49.580*** (13.251)	6.677 (19.232)	43.649*** (14.606)
GDP^2^	− 0.515*** (0.153)	− 6.752*** (1.821)	− 0.576 (2.344)	− 4.201*** (1.431)
GDP^3^	0.016*** (0.006)	0.309*** (0.083)	0.015 (0.095)	0.135*** (0.047)
REN	− 0.258*** (0.012)	− 0.478*** (0.017)	− 0.411*** (0.025)	− 0.142*** (0.016)
R&D	0.077*** (0.008)	0.217*** (0.013)	0.110*** (0.014)	0.057*** (0.010)
TRD	0.262*** (0.030)	− 0.122** (0.053)	0.371*** (0.040)	0.151*** (0.043)
INS	− 0.011* (0.006)	− 0.062*** (0.008)	0.037*** (0.009)	− 0.006 (0.026)
Intercept	− 21.290	− 121.340	52.416	− 150.600
Observations	1045	171	323	551
Countries	55	9	17	29
*R* ^2^	0.820	0.956	0.692	0.527
Adjusted *R* ^2^	0.819	0.954	0.685	0.521

***, **, and * indicate significant *p* values at the 1, 5, and 10% level, respectively. Standard errors are presented in the parentheses

**Table 13:** Pooled OLS when excluding REN

Explanatory variables	Total sample	Lower MIC	Upper MIC	HIC
GDP	1.574 (1.333)	28.976 (25.769)	− 17.185 (25.607)	32.389*** (11.145)
GDP^2^	0.010 (0.153)	− 4.011 (3.625)	2.413 (3.123)	− 3.109*** (1.106)
GDP^3^	− 0.005 (0.006)	0.189 (0.169)	− 0.107 (0.127)	0.100*** (0.036)
R&D	0.128*** (0.009)	0.297*** (0.023)	0.083*** (0.018)	0.069*** (0.009)
TRD	0.414*** (0.030)	0.575*** (0.093)	0.565*** (0.051)	0.157*** (0.034)
INST	− 0.043*** (0.005)	− 0.057*** (0.014)	− 0.054 (0.009)	− 0.044*** (0.007)
Intercept	− 12.274	− 75.068	36.283	− 111.836
Observations	1406	323	380	703
Countries	74	17	20	37
*R* ^2^	0.754	0.563	0.472	0.394
Adjusted *R* ^2^	0.753	0.555	0.464	0.389

***, **, and * indicate significant *p* values at the 1, 5, and 10% level, respectively. Standard errors are presented in the parentheses

**Table 14:** VIF test

Variables	VIF
GDP	3.123
REN	1.554
R&D	1.536
TRD	1.368
INS	2.655

## Footnotes

1 ASEAN-5 includes Indonesia, Malaysia, the Philippines, Singapore, and Thailand (Duan et al. 2016) and APEC stands for Asia-Pacific Economic Cooperation (Zhang et al. 2016).

2 See, e.g., Lee et al. (2009), Al-Mulali and Ozturk (2015), You et al. (2015), Al-Mulali et al. (2016), Friedl and Getzner (2003), Duan et al. (2016), Nguyen et al. (2017), and Sohag et al. (2017).

3 In the political rights index, the functioning of the government, electoral process, and political pluralism and participation are included. Associational and organizational rights, personal autonomy and individual rights, freedom of expression and belief, and the rule of law are included in the civil liberties index. As the ideas about civil liberties and political rights constantly evolve changes in the methodology are sometimes made. However, when changes are made, they are introduced gradually, so the comparability between the years remain possible (Freedom House 2017b). We add up these indexes so that 13 is the highest level of institutional quality.
